# Silver-Based Chemical Device as an Adjunct of Domestic Oral Hygiene: A Study on Periodontal Patients

**DOI:** 10.3390/ma11081483

**Published:** 2018-08-20

**Authors:** Valentina Candotto, Dorina Lauritano, Francesco Carinci, Carlo Alberto Bignozzi, Daniele Pazzi, Francesca Cura, Marco Severino, Antonio Scarano

**Affiliations:** 1 Department of Biomedical, Surgical and Dental Sciences University of Milan, 20122 Milan, Italy; valentina.candotto@student.unife.it; 2 Department of Medicine and Surgery, Centre of Neuroscience of Milan, University of Milan-Bicocca, 20900 Milan, Italy; dorina.lauritano@unimib.it; 3 Department of Morphology, Surgery and Experimental Medicine, University of Ferrara, 44121 Ferrara, Italy; crc@unife.it; 4 Department of Chemical and Pharmaceutical Sciences, University of Ferrara, 44121 Ferrara, Italy; g4s@unife.it (C.A.B.); pazzidaniele@gmail.com (D.P.); 5 Department of Experimental, Diagnostic and Specialty Medicine, University of Bologna, 40126 Bologna, Italy; cura.francesca@tiscali.it; 6 Department of Life, University of Aquila, 67100 L’Aquila, Italy; marco.severino@univaq.it; 7 Department of Oral Science, Nano and Biotecnology and CeSi-Met University of Chieti-Pescara, 66100 Chieti, Italy

**Keywords:** chronic periodontitis, bacterial load, oral biofilm, red complex

## Abstract

The use of chemical devices for periodontitis treatment has led to new strategies aiming primarily to control infections. Over the last few years, new chemical devices have been subjected to many scientific and medical studies. The purpose of the present study was to assess the effect of a new silver based chemical devices gel named “Hydrosilver Plus Gel”, abbreviated here as Hydrosilver, on the pathogenic microorganisms, using Polymerase Chain Reaction (PCR) for microbiological analysis. *Materials and methods*: Ten patients with a diagnosis of chronic periodontitis in the age group >25 years were selected. None of these patients had received any surgical or non-surgical periodontal therapy, and demonstrated radiographic evidence of moderate bone loss. After scaling and root planning, patients received Hydrosilver to be used at home. Four non-adjacent sites in separate quadrants were selected in each patient for monitoring, based on criteria that the sites localise chronic periodontitis. Microbial analysis was analysed at baseline and at Day 15. SPSS program was used for statistical purposes and a paired samples correlation was performed at the end of the observation period. *Results*: Mean amounts of bacterial loading before and after Hydrosilver treatment reduced statistically significantly (*p* = 0.002). *Conclusions*: The present study demonstrated that Hydrosilver has a good impact on oral biofilm. Additional studies are needed to detect the efficacy of this chemical device.

## 1. Introduction

Periodontal disease (PD) is supposed to be the leading cause of tooth loss in the adult population [1]. PD is an inflammatory disease characterised by the formation of pathogenic microorganisms (PMs) on both the tooth surfaces and gingival tissues. Thousands of different species of PMs inhabit the oral cavity, of which 150–200 are found in most individuals and over 400 are found in the periodontal pocket [2]. Theses PMs involve quorum sensing (cell-to-cell communication), metabolic cooperation, and show ability to elude the host immune system causing a high resistance to antibiotics [3].

The rapid innate immune system of the oral epithelia, gingival crevicular fluid, and saliva form the first line of defence against colonisation by PMs, that can disrupt the membrane integrity, targeting the cytoplasmic elements, ultimately causing bacterial cell death. Hence, oral microbiota help maintain the complex ecological homeostasis between the host and PMs in the oral environment [4].

The oral cavity is unique: the biofilm and PMs have easy access to the rest of the body through the epithelium and the gastrointestinal tract [4]. The gingival epithelial cells, on exposure to PMs related to PD, actively express defence mechanisms. Nearly twenty genetic disorders connected to PD have been identified so far and are associated with the expression of host defence mechanisms [5].

The gingival epithelium possesses a broad spectrum of activity against both Gram-positive and Gram-negative bacteria including PMs such as *Porphyromonas gingivalis*, *Prevotella intermedia*, and *Aggregatibacter actinomycetemcomitans* alongside fungi and viruses. It miraculously neutralises the activity of lipopolysaccharides, helping to protect the tissues from their harmful effects. They maintain a balance between pro- and anti-inflammatory mediators at the time of lipopolysaccharides attack [5].

The onset of PD is related to a microbial shift of PMs, more commonly known as dysbiosis, due to a change in the proportion of saprophytes and PMs. PD is the result of changes in biofilm community of endogenous species. Although the pathogens of PD have been studied from a long time, the identification of the PMs is difficult. However, it is now clear that bacteria of “red complex” type are the main PMs. The “red complex” bacteria (*Porphyromonas gingivalis*, *Tannerella forsythia*, and *Treponema denticola*) are widely considered as major periodontal PMs. Members of the orange complex (*Fusobacterium nucleatum* and *Campylobacter rectus*) as well as *Aggregatibacter actinomycetemcomitans*, *Atopobium rimae*, *Eubacterium saphenum*, *Porphyromonas endodontalis*, and *Treponema lecithinolyticum* are also considered potentially pathogenic species [6]. In addition, *Capnocytophaga ochracea* was suggested as a diagnostic marker of periodontal health [7].

Non-surgical periodontal therapy is widely accepted as a method to keep the natural teeth and preserve oral health, and home oral hygiene is also very important [8]. The objective of home oral hygiene consists in the elimination of infection and prevention of disease progression. It is now well established that prevention consists in the control of PMs, in supportive periodontal therapy, and that the maintenance of oral health is related to proper prevention care programmes. Although preventive protocols are demonstrated to be widely effective, periodontal disease may present relapse caused by poor oral hygiene. The use of topical antimicrobials has proven to be effective for the treatment of periodontal disease in addition to non-surgical periodontal therapy. In particular, nano-material has much potential in dentistry due to difficulties with antibiotic resistance [9,10,11,12]. These nanoparticles show a synergistic antibacterial and antibiofilm effects in clinical and standard strains [11]. Different local drug delivery systems with chemical devices have been introduced; used to the base of the pocket, they minimise the adverse impact on non-oral body sites [13,14,15,16,17,18].

### Aim

The aim of this study was to assess the effect of a new oral gel named “Hydrosilver Plus Gel”, abbreviated here as Hydrosilver, on oral bacterial loading using Polymerase Chain Reaction (PCR).

## 2. Materials and Methods

### 2.1. Gel Composition

The gel contains a 0.1% w/w of silver ions, Ag^+^, a 0.14% of thiosalicycilic acid, sodium salt and a 1.6% of sodium hyaluronate swollen in water. The silver ions are bound to the salicylate anion to form a negatively charged complex, which is water soluble and compatible with the presence of sodium hyaluronate.

### 2.2. Experimental Design and Patient Selection

Ten patients with a diagnosis of chronic periodontitis, >25 years age, were selected. None of these patients had received any surgical or non-surgical periodontal therapy and demonstrated radiographic evidence of moderate bone loss. Four non-adjacent sites in separate quadrants were selected in each patient for monitoring, based on criteria that the sites localised chronic periodontitis. Microbial analysis was performed at baseline and on Day 15.

During the initial phase, all the subjects were trained about proper home-care techniques and monitored during the study period, receiving full mouth scaling and root planning. All subjects received microbiological and clinical monitoring at baseline and after 15 days, respectively. The research objectives were explained to the patients, who then signed an informed consent form. This study was conducted in accordance with the Declaration of Helsinki, and the protocol was approved by the Ethics Committee of 06.09.2013 prot. n. 29579 University Study of L’Aquila.

### 2.3. Subject Population and Inclusion/Exclusion Criteria

Ten subjects with untreated chronic periodontitis were selected by one of the authors. Detailed medical, periodontal and dental history was obtained. All eligible subjects were informed of the nature, potential risks and benefits of the study. The inclusion criteria were as follows: age >25 years; probing depth of 3 mm or more; and dentate with >20 natural teeth. The exclusion criteria were: medically compromised patients; patients who had been administered antibiotics or antimicrobial in the past 6 months; smokers; and pregnant and lactating mother.

### 2.4. Determination of Periodontal Status

All participants were evaluated clinically. The same examiner assessed the clinical and periodontal parameters. Probing depth measured to the nearest millimetre from the gingival margin to the bottom of the pocket was noted using calibrated William’s periodontal probe for all measurements.

### 2.5. Microbiological Test

The antimicrobial activity of the gel was tested in vitro against Gram-positive and Gram-negative bacteria and *Candida albicans*. A remarkable inhibition ring is produced by 50 μL of Hydrosilver placed in the centre of a PETRI capsula containing TSA (Tripton Soia Agar) which was previously contaminated on the surface with 100 μL of a microbic pool containing *Staphylococcus aureus* ATCC 6538, *Escherichia coli* ATCC 10536, *Pseudomonas aeruginosa* ATCC 15442, *Enterococcus hirae* ATCC 10541 and *Candida albicans* ATCC 1023 in concentration of the order of 1.5–5 × 10^10^ UFC/mL (Diagnostic International Distribution S.p.A, Milan, Italy).

In addition to these experiments, LABtest^®^ (LAB SRL^®^, Ferrara, Italy) was used [19]. It detects Aggregatibacter actinomycetemcomitans, Porphyromonas gingivalis, Tannerella forsythia, Treponema denticola, Fusobacterium nucleatum, Campylobacter rectus and total bacteria loading.

### 2.6. Real-Time Polymerase Chain Reaction

Primers and probes oligonucleotides were designed based on 16S rRNA gene sequences of the Human Oral Microbiome Database (HOMD 16S rRNA RefSeq Version 10.1) counting 845 entries. All the sequences were aligned to find either consensus sequence or less conservate spots. Three real-time polymerase chain reaction (PCR) runs were performed for each sample. The first reaction quantifies the total amount of bacteria using two degenerate primers and a single probe matching a highly conservated sequence of the 16S ribosomal RNA gene. The second reaction detects and quantifies the three red complex bacteria, i.e., *Porphyromonas gingivalis*, *Tannerella forsythia* and *Treponema denticola*, in a multiplex PCR. The third reaction detects and quantifies *Aggregatibacter actinomycetemcomitans*, *Fusobacterium nucleatum* and *Campylobacter rectus*. These reactions include six primers each and three probes each that were highly specific for each specie. Oligonucleotide concentrations and PCR conditions were optimised to ensure sensitivity, specificity and no inhibition in the case of unbalanced target amounts. Absolute quantification assays were performed using the Applied Biosystems 7500 Sequence Detection System (Thermofisher Scientific, Monza, Italy)。 The amplification profiles were initiated by a 10 min incubation period at 95 °C to activate polymerase, followed by a two-step amplification of 15 s at 95 °C and 60 s at 57 °C for 40 cycles. All experiments were performed including non-template controls to exclude reagents contamination.

### 2.7. Agents (S1) 125

Plasmids containing synthetic DNA target sequences (Eurofin MWG Operon, Ebersberg Germany) were standardly used for the quantitative analysis. Standard curves for each target were constructed in a triplex reaction by using a mix of the same amount of plasmids in serial dilutions ranging from 10 to 10^7^ copies. There was a linear relationship between the threshold cycle values plotted against the log of the copy number over the entire range of dilutions (data not shown). The copy numbers for individual plasmid preparations were estimated using the Thermo NanoDrop spectrophotometer (Thermofisher Scientific, Monza, Italy).

The absolute quantification of total bacterial genome copies in samples allowed for the calculation of relative amount of specific bacterial species. To prevent samples and polymerase chain reaction contamination, plasmid purification and handling were performed in a separate laboratory with dedicated pipettes.

### 2.8. Statistical Analysis

Descriptive statistics were performed using Microsoft Excel 2016 spreadsheets. Paired *t*-test from SPSS program was used to statistically evaluate the change in specific bacteria loading before and after treatment.

## 3. Results

Mean amounts of bacterial loading before and after Hydrosilver treatment were reduced in most cases (Table 1). Paired sample correlations demonstrated a reduction of TBL after therapy (total bacteria loading) (*p* = 0.002) (Table 2).

## 4. Discussion

In the oral cavity, bacterial infection induces an inflammatory response with progressive destruction of the periodontal tissues and finally the loss of teeth. The definition of periodontitis has changed considerably over the years [20]. Specific PMs of the biofilm formed by plaque cause periodontitis. The PMs leak into the periodontal ligament space, causing anaerobic infection creating a cascade of events, which ends with the production of inflammatory mediators and bacterial metabolites. PD affects about the 50% of adults or more, while; the severe periodontitis form affects around 10% (range: 5–20%) of adults and moderate periodontitis around 30%. In elderly people aged 60–74, the prevalence of PD is estimated at about 70–90%, aged from 60- to74-years-of-age [21].

The PMs associated with periodontal diseases are susceptible to a variety of antiseptics and antibiotics. Non-surgical periodontal therapy, associated with proper domestic oral hygiene, has been documented to preserve the natural dentition by achieving and maintaining a healthy periodontium [22]. According to the current state of knowledge, species such as the red complex organisms are just a few of those PMs that have been shown to play a major role in the pathogenesis of PD [6].

PMs are also the main cause of peri-implantitis. In fact, dental implants have had great success in the last decades for replacing missing teeth in partially or totally edentulous patients. Even if the main factor for implant dentistry success is the quality of bone of receiving sites, PMs also cause peri-implantitis [23].

In this study, a strong reduction of total bacterial loading using the Hydrosilver for domestic oral hygiene was observed. The reduction of total bacteria can be attributed to the domestic use of the gel and its antibacterial properties.

Since there was no control group, it is difficult to draw final conclusions about the clinical efficiency of the Hydrosilver, although the reported data show a drop in total bacterial loading after treatment. It is our knowledge that a randomised controlled trial is mandatory to have final proof about the efficacy of the new molecule to reduce bacterial loading inside periodontal pocket.

The oral cavity is the interface between the human body and the external world. The external layer of oral epithelium is composed of various epithelia, which constitute barriers guaranteeing the integrity of the organism and protecting it from invasion by PMs. It is well known that PD is pathology-associated with PMs. Genetic differences in oral plaque, background and general health of patients can control the development of PD [6].

The association of PD and systemic diseases, such as diabetes, cardiovascular diseases and pre-term birth, reveals the underestimated importance of this disease for global health. A thorough and early diagnosis of PD allows a more accurate risk calculation for developing systemic pathologies [24]. If a causative relationship is established between PD and these pathologies, therapeutic management of PD will become part of their prevention [24,25].

## 5. Conclusions

A wide range of PMs has been discovered in various niches in the oral cavity. Their potential roles in regulating the oral environment in both health and disease have also been successfully described in the literature. They have been conserved in evolution and show relatively higher potential to PD onset thus potentially deregulate the biofilm environment and progressing gingival and periodontal diseases. Not just this but the role of PMs in controlling the spread of caries has also been documented. Challenges such as their design, synthesis, and function at molecular level need to be overcome in the near future to open doors toward the design of potentially effective oral microbial antibiotics.

The results of the study provide preliminary data on an overall reduction in bacterial loading with use of Hydrosilver (*p* = 0.002).

Since there was no control group, it is difficult to draw final conclusions about the clinical efficiency of the Hydrosilver, although the reported data show a statistically significant drop in total bacterial loading after treatment.

We believe a randomised controlled clinical trial is mandatory to have final proof about the efficacy of the new molecule to reduce specific and total bacterial loading inside periodontal pocket.

## Figures and Tables

**Table 1:** Mean amounts of specific bacterial species before and after Hydrosilver treatment rounded to 1 significant figure AA, *Aggregatibacter actinomycetemcomitans*; CR, *Campylobacter rectus*; FN, *Fusobacterium nucleatum*; PG, *Porphyromonas gingivalis*; TBL, total bacteria loading; TD, *Treponema denticola*; TF, *Tannerella forsythia*; 1, before; 2, after treatment.

Comparison between Specific Bacteria before and after Treatment	Bacteria Species before and after Treatment	Mean ± (SD)
Pair 1	AA1	2000 ± 4000
	AA2	500 ± 1000
Pair 2	CR1	600 ± 800
	CR2	2000 ± 6000
Pair 3	FN1	20,000 ± 40,000
	FN2	7000 ± 10,000
Pair 4	PG1	100 ± 300
	PG2	1 ± 0.1
Pair 5	TBL1	300,000 ± 2,000,000
	TBL2	800,000 ± 1,000,000
Pair 6	TD1	200 ± 500
	TD2	9 ± 30
Pair 7	TF1	1000 ± 2000
	TF2	90 ± 200

**Table 2:** Samples T test output.

Comparison between Specific Bacteria before and after Treatment	Bacteria Species before and after Treatment	Mean Value and Standard Deviation	95% Confidence Interval of the Difference		
		Mean ± SD	Lower	Upper	Sig.
Pair 1	AA1-AA2	2000 ± 4000	−1000	4000	2
Pair 2	CR1-CR2	−1000 ± 6000	−6000	3000	0.5
Pair 3	FN1-FN2	10,000 ± 40,000	−10,000	40,000	0.3
Pair 4	PG1-PG2	100± 300	−80	300	0.2
Pair 5	TBL1-TBL2	2,000,000 ± 2,000,000	1,000,000	3,000,000	0.002
Pair 6	TD1-TD2	200 ± 500	−100	600	0.2
Pair 7	TF1-TF2	1000 ± 2000	−700	3000	0.3
